# New Pathogenic Germline Variants in Very Early Onset and Familial Colorectal Cancer Patients

**DOI:** 10.3389/fgene.2020.566266

**Published:** 2020-09-24

**Authors:** Malene Djursby, Majbritt B. Madsen, Jane H. Frederiksen, Lukas A. Berchtold, Christina Therkildsen, Gro L. Willemoe, Jane P. Hasselby, Friedrik Wikman, Henrik Okkels, Anne-Bine Skytte, Mef Nilbert, Karin Wadt, Anne-Marie Gerdes, Thomas van Overeem Hansen

**Affiliations:** ^1^Department of Clinical Genetics, Rigshospitalet, Copenhagen University Hospital, Copenhagen, Denmark; ^2^Center for Genomic Medicine, Rigshospitalet, Copenhagen University Hospital, Copenhagen, Denmark; ^3^The Danish HNPCC Register, Clinical Research Centre, Copenhagen University Hospital, Hvidovre, Denmark; ^4^Department of Pathology, Rigshospitalet, Copenhagen University Hospital, Copenhagen, Denmark; ^5^Department of Molecular Medicine, Aarhus University Hospital, Aarhus, Denmark; ^6^Section of Molecular Diagnostics, Department of Clinical Chemistry, Aalborg University Hospital, Aalborg, Denmark; ^7^Department of Clinical Genetics, Aarhus University Hospital, Aarhus, Denmark

**Keywords:** hereditary colorectal cancer, gene panel analysis, familial cancer, oligogenic inheritance, early onset colorectal cancer, *RPS20*

## Abstract

A genetic diagnosis facilitates personalized cancer treatment and clinical care of relatives at risk, however, although 25% of colorectal cancer cases are familial, around 95% of the families are genetically unresolved. In this study, we performed gene panel analysis on germline DNA of 32 established or candidate colorectal cancer predisposing genes in 149 individuals from either families with an accumulation of colorectal cancers or families with only one sporadic case of very early onset colorectal cancer (≤40 years at diagnosis). We identified pathogenic or likely pathogenic genetic variants in 10.1% of the participants in genes such as *APC*, *POLE*, *MSH2* or *PMS2*. The *MSH2* variant, c.2168C>T, p.(Ser723Phe) was previously described as a variant of unknown significance, but we have now reclassified it to be likely pathogenic. The *POLE* variant, c.1089C>A, p.(Asn363Lys) was identified in a patient with three metachronous colorectal cancers from age 28 and turned out to be *de novo*. One pathogenic *PMS2* variant was novel. We also identified a number of highly interesting variants of unknown significance in *APC*, *BUB1, TP53* and *RPS20*. The *RPS20* variant is novel and was found in a large Amsterdam I positive family with a multi tumor phenotype including 12 cases of CRC from as early as age 24. This variant was found to segregate with cancer in the family and multiple *in silico* tools predict it to be pathogenic. Our data further support the shift from phenotypic-based cancer panels to large panels including all established genes involved in hereditary cancer syndromes or (targeted) whole genome sequencing. Additionally, identification of a likely disease-predisposing variant in *RPS20* expands the phenotypic spectrum of *RPS20*-related cancers and emphasize that this gene is relevant to include in colorectal cancer gene panels.

## Introduction

Colorectal cancer (CRC) is one of the most frequent types of cancer worldwide and is now the second most common cancer in Denmark. Approximately 20% to 30% of the cases report a family history with other cases of CRC, however, in more than 95% of the affected cases a genetic etiology cannot be identified (Schubert et al., 2019). The families are highly heterogeneous regarding phenotypes, inheritance patterns and overall lifetime cancer risk, making genetic counseling and surveillance a challenge. An established genetic diagnosis facilitates personalized cancer treatment, and surveillance of affected and unaffected carriers, emphasizing the importance of identifying the genetic background of the disease.

Traditionally, Danish patients or families suspected of having hereditary CRC, for example due to familial aggregation of CRC, early-onset disease or multiple primary hereditary non-polyposis colorectal cancer (HNPCC) associated tumors, have been offered genetic counseling and genetic test of the Lynch Syndrome-predisposing mismatch repair (MMR) genes (*MLH1, MSH2, MSH6, PMS2*) and *EPCAM*, and in case of several colonic adenomas in addition *APC* and *MUTYH*. In the past 5 years a larger gene panel consisting of 17 CRC-predisposing genes (*APC, AXIN2, BMPR1A, EPCAM, GREM1, MLH1, MSH2, MSH3, MSH6, MUTYH, NTHL1, PMS2, POLD1, POLE, PTEN, SMAD4* and *STK11)* has usually been analyzed, however, the great majority of cases are still genetically unresolved.

Lack of a genetic diagnosis is a worldwide issue in familial and early onset CRC and has led to many studies utilizing larger cancer panels in search of genetic explanations. The number of analyzed genes has varied, but depending on the cohort and previous genetic analyses, a monogenetic etiology – often including variants in genes with uncertain clinical impact and low- or moderate risk variants – has been identified in up to 22% of the patients analyzed, with the highest diagnostic yield in younger patients (Chubb et al., 2015; Mork et al., 2015; Hansen et al., 2017; Pearlman et al., 2017; Yurgelun et al., 2017; Dominguez-Valentin et al., 2018; Martin-Morales et al., 2018; Stoffel et al., 2018). However, some studies have shown a very limited diagnostic yield when analyzing well-established cancer predisposition genes suggesting that other genetic inheritance patterns or mechanisms should be sought. Several possible disease-causing genetic mechanisms have been proposed including variants in not yet identified highly penetrant cancer genes, mosaicism, regulatory- and deep intronic variants in known cancer genes, epigenetic alterations, or di-, oligo- or polygenic inheritance (Schubert et al., 2019); further studies exploring these mechanisms are warranted.

In this study, we aimed at identifying rare or novel germline variants in 32 established or suggested cancer predisposition genes in a cohort of highly selected Danish patients with either very early onset sporadic CRC (i.e., ≤40-years-old) or in families with familial CRC and without identified MMR-deficiency.

## Materials and Methods

### Patients

The participants were recruited from two cohorts: (1). Families with familial CRC (the ‘Familial CRC cohort’) and (2). Families with only one case of early onset CRC (the ‘Early onset CRC cohort’). Family data was extracted from the Danish Hereditary Non-Polyposis Colorectal Cancer (HNPCC) registry (Clinical Research Centre, Copenhagen University Hospital, Hvidovre, Denmark). The registry covers all parts of Denmark and has, since 1991, records of all families with, or suspected of having, hereditary CRC. In addition, some patients/families were identified through genetic counseling in Department of Clinical Genetics, Rigshospitalet, and invited/included in the study. They fulfilled the same inclusion criteria. For both cohorts, we included patients without known Lynch Syndrome, or not previously tested. Patients/families with previously identified variants of unknown significance in cancer genes were kept in the study in order to search for alternative explanations. Previous identification of pathogenic variants in other CRC-predisposing genes caused exclusion.

**FIGURE 1:**
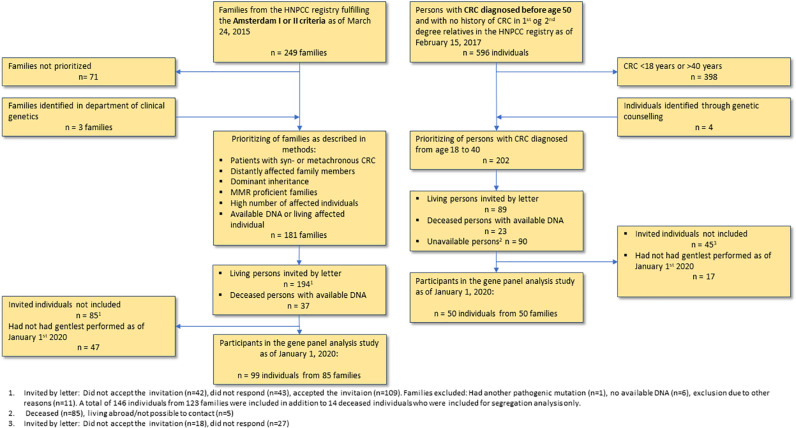
Flow diagram of the inclusion process.

The patients included in this study had gene panel analyses performed by January 1st, 2020. Flow diagram of the inclusion process can be found in Figure 1.

### The Familial CRC Cohort

We received data on all Amsterdam I or II positive families (i.e., families with at least three cases of CRC (= Amsterdam I) or HNPCC-associated cancers (cancer of the endometrium, small intestine, ureter or renal pelvis = Amsterdam II), affecting at least two successive generations, with one relative diagnosed before the age of 50 years; one should be a first-degree relative of the other two and familial adenomatous polyposis (FAP) should be excluded) without, or not tested for, Lynch Syndrome. A total of 249 families fulfilled the search criteria as of March 24th, 2015. Based on pedigrees, pathology reports and previous molecular analyses, we selected the families most likely to have a monogenic, non-MMR high risk variant, i.e., families with a high number of affected individuals with CRC (preferably synchronous/metachronous CRC), multiple primary cancers or colonic adenomas (preferably advanced adenomas, i.e., size ≥ 10 mm., with high grade dysplasia or villous/tubulovillous morphology), young age at onset and a clearly dominant inheritance pattern without unaffected generations, and with available DNA or a living affected individual. A total of 181 families were selected for inclusion. All recruited subjects had to fulfill our inclusion criteria by having (1). Any type of cancer or (2). Colonic adenomas (either ≥3 colorectal adenomas or ≥1 advanced, colorectal adenoma).

### The Early Onset CRC Cohort

We received data on all families with only a single case of CRC before age 50, and without a family history of CRC in first degree relatives and grandparents (*n* = 596 patients as of February 15th, 2017). The search criteria only included patients without genetically identified Lynch Syndrome, or not previously tested. Patients with CRC between 18 to 40 years at time of diagnosis (*n* = 198) were recruited.

### Inclusion and Follow Up

After updating the pedigree with relevant clinical information, some families did not fulfill the inclusion criteria. In the early onset CRC cohort this could be due to a newly developed case of CRC in the family, resulting in CRC in a first or second degree relative, and in the familial CRC cohort it could be due to original inadequate family data, such as a case of polyposis not reported to the registry. Since family history is a process of constant development, we kept these families in the study.

All living patients received written and oral information as well as genetic counseling, and a written informed consent was obtained. Ethical approval was obtained from the Danish Committee on Health Research Ethics (reference: H-4-2014-050). In total, we included 149 individuals: 50 patients with early onset CRC and 99 patients from 85 families with familial CRC. Characteristics of the participants are shown in Table 1.

**TABLE 1:** Clinical characterization of the participants.

	**All *N* = 149**	**Early onset CRC cohort *N* = 50**	**Familial CRC cohort *N* = 85**
Number of participants	149	50	99
Number of families	135	50	85
Amsterdam I positive	75	–	75
Amsterdam II positive	9	–	9
Do not fulfill Amsterdam I/II criteria	1	50	1
**Gender and age at diagnosis**			
Male	67	23	44
Female	82	27	55
Median age at first diagnosis (range)	46 years (22–78)	36 years (28–40)	52 years (22–78)
**Results of genetic testing**			
Number of patients/families with pathogenic/likely pathogenic variants (%)	15/149 (10.1%)	5/50 (10%)	9/85 (10.6%)
Number of clinically actionable variants	6	3	3
Number of patients/families with clinically actionable variants (%)	8 (5.4%)	4 (8%)	3 (3.5%)
**Cancer diagnosis**			
Right-sided colon cancer^1^	46	17	29
Left-sided colon cancer^2^	55	15	40
Rectal cancer^3^	46	16	30
Colorectal cancer, unspecified	5	3	2
Gynecological cancer^4^	7	0	7
Breast cancer^5^	3	0	3
Other gastrointestinal cancers^6^	5	1	4
Other cancers^7^	7	1	6
Only adenomas^8^	2	0	1
Synchronous/metachronous CRC	13	1	12
Multiple (i.e., >3) primary tumors	3	0	3
**Polyps**			
No adenomas detected	91	43	48
1–9 adenomas	53 (median 2)	7 (median 1)	46 (median 2,5)
>10 colonic adenomas^9^	3	0	2
Number of high-grade dysplastic adenomas	5	1	4
>10 serrated lesions	4	1	3
Has ≥1 relative(s) with >10 colonic adenomas	5	0	4
**Tumor data**			
Number of patients/families with IHC analysis performed	118/149 (79.2%)	45/50 (90%)	73/85 (85.9%)
Percentage of patients/families with abnormal IHC	12.7% (15/118)	17.8% (8/45)^10^	9.6% (7/73)^11^
**Previous genetic analyses at time of inclusion**			
MMR analysis in the index person	109 (73.2%)	41 (82%)	68 (68.7%)
No previous MMR analysis in the index person or a relative	16 (10.7%)	9 (18%)	7 (8.2%)
**Family history**			
≥1 sibling(s) or child(ren) with CRC <50 years (%)	25	0	25
≥1 sibling(s) or child(ren) with any cancer <60 years	46	1	43
Both parents diagnosed with CRC (%)	6	0	6
Mean number of persons with CRC in the family (1st–3rd degree relatives (range)	2.8 (1–7)	1.06 (1–2)	3.7 (1–7)

*Cases of basal cell carcinomas were not included in this table. 1: Adenocarcinomas located in cecum, appendix, ascending- or transverse colon. 2: Adenocarcinomas located in the splenic flexure, descending colon or sigmoid colon. 3: Only adenocarcinomas. 4: One vulvar cancer (no other cancers), four endometrial cancers (two persons also had CRC, one person also had CRC and breast cancer and one person also had thyroid cancer) and two ovarian cancers (one also had CRC and breast cancer). 5: One person also had CRC, one person also had endometrial cancer and one person also had both CRC and ovarian cancer. 6: One duodenal cancer, one ileal cancer, one esophageal cancer (squamous carcinoma), one carcinoma at the major duodenal papilla, one hepatic cancer (a hepatocellular carcinoma). All patients also had CRC except the patient with duodenal cancer, who had >100 colonic adenomas. 7: Two persons had bladder cancer, one person had prostate cancer, one person had chronic lymphatic leukemia and one person had melanoma; they all also had CRC. One person had thyroid cancer and endometrial cancer. 8: Another person from the same family with three metachronous CRCs were also included. 9: Two persons from the same family had >100 and 25 colonic adenomas, respectively. Another person had 20 colonic adenomas. 10: Two persons with lack of PMS2 expression had pathogenic *PMS2* variants. 11: In seven persons, IHC analysis revealed absence of one or more MMR-proteins, however, they all had a relative with a normal IHC analysis. Two persons had *MLH1* promoter methylation.*

### Next Generation Sequencing Analysis

#### DNA Extraction

Genomic DNA was extracted from whole blood samples using ReliaPrep Large Volume HT gDNA Isolation Kit (Promega, Madison, WI, United States) using a Tecan Freedom EVO HSM2.0 Workstation according to the manufacturer’s instructions.

#### Sequencing

The following 32 genes were examined by next-generation sequencing (NGS): *APC* (NM_000038), *AXIN2* (NM_004655), *BLM* (NM_000057), *BMPR1A* (NM_004329, *BRCA1* (NM_007294, *BRCA2* (NM_000059), *BUB1* (NM_004336), *CDH1* (NM_004360), *CHECK2* (NM_007194), *EXO1* (NM_006027), *FAN1* (NM_014967), *FOCAD* (NM_017794), *GALNT12* (NM_024642), *IPMK* (NM_152230), *MLH1* (NM_000249), *MLH3* (NM_014381), *MSH2* (NM_000251), *MSH3* (NM_002439), *MSH6* (NM_000179), *MUTYH* (NM_001128425), *NTHL1* (NM_002528), *PMS1* (NM_000534), *PMS2* (NM_000535), *POLD1* (NM_002691), *POLE* (NM_006231), *PTEN* (NM_000314), *RINT1* (NM_021930), *RPS20* (NM_001146227), *SMAD4* (NM_005359), *SMAD9* (NM_001127217), *STK11* (NM_000455), *TP53* (NM_000546). Target DNA sequences were captured using biotinylated oligos provided through Roche NimbleGen (Roche, Basel, Switzerland). The oligos were designed to capture all exons, including 50 bp of flanking intronic sequence. Library was constructed using 1400 ng of genomic DNA. The DNA was fragmented into an average size of 400 bp using a Covaris S2 AFA ultrasonicator. The trimming, 3′-adenylation and adaptor ligations were done on a Sciclone G3robot (Perkin Elmer, Waltham, MA, United States) using Illumina-compatible KAPA library DNA adaptors (Roche Diagnostics, Basel, Switzerland). Sequence capture was performed using the single capture protocol as described by Roche NimbleGen, where 6 to 12 samples are multiplexed before hybridization. Finally, 2 × 151-bp paired-end sequencing was performed on the Illumina MiSeq platform to an average depth of >50X (range 54.2–5893.4X) with a coverage of at least 20X in >98% of the targeted nucleotides and 30X in >97% of the targeted nucleotides.

#### Data Processing

Sequencing reads were trimmed and mapped to human reference genome hg19/GRCh37 using BWA-MEM v0.7.15 software (Li, 2013). Alignment quality control was performed with mosdepth v0.2.4 (Pedersen and Quinlan, 2018) for all target regions. Using GATK v4.1.0.0 suite (McKenna et al., 2010) alignment files were pre-processed and germline variants were called (Poplin et al., 2017). Variant files were filtered to exclude variants covered by <10 reads or called in <20% of the sequencing reads.

#### Ingenuity Variant Analysis

Called variants were filtered using Ingenuity Variant Analysis (IVA^1^). Firstly, variants with call quality < 20, read depth < 10 or variant allele frequency (VAF) < 15 were disregarded. Secondly, variants with an allele frequency > 5% of the public variant database including 1000 genomes project^2^, ExAC^3^, gnomAD^4^ or NHLBI ESP exomes^5^, unless established as a pathogenic common variant, were excluded. Variants with a minor allele frequency (MAF) between 0.5% and 5% in any subpopulation in gnomAD were not further analyzed unless a class 4 or 5 variant were detected in a patient in a gene known to cause autosomal recessive cancer. Thirdly, variants in coding regions (including missense variants regardless of *in silico* prediction and synonymous variants) and splice-site variants (±10 bp) were kept for further analysis, as well as variants listed in ClinVar, with gain of function established in the literature or with a CADD score > 20.

#### Variant Classification

All variants identified after IVA processing were reviewed and classified according to the ACMG-AMP guidelines (Richards et al., 2015). We used the following five-class system: 1 = Not pathogenic/no clinical significance, 2 = Likely not pathogenic/little clinical significance, 3 = Uncertain, 4 = Likely Pathogenic, 5 = Pathogenic (Plon et al., 2008). Variant classification by expert panels such as ENIGMA^6^ or InSiGHT^7^ were followed unless new knowledge had emerged. All variants were analyzed manually and evaluated using different tools or databases such as Alamut Visual^8^ including *in silico* splice prediction, LOVD^9^, ClinVar^10^, COSMIC^11^ and literature search in PubMed^12^.

#### Validation of Variants

All pathogenic and likely pathogenic variants as well as all variants in Table 3 were validated by visual inspection using the Integrative Genomics Viewer^13^ (Robinson et al., 2011), and all variants used in a clinical setting were validated from a new blood sample, either by NGS analysis or by Sanger sequencing.

### RPS20 Segregation Analysis

In one family member from family 92, the *RPS20* c.98A>T variant was identified in a clinical setting in another department of clinical genetics during the time our study was running. In the rest of the tested family members, *RPS20* c.98A>T segregation analysis was performed by Sanger sequencing (primer sequences are available upon request) when possible. For some of the family members only non-malignant formalin-fixated paraffin-embedded (FFPE) tissue samples were available, and some of those were of a quality that did not allow Sanger sequencing, so NGS was used instead. One tissue sample failed with both methods.

### Long Range PCR

For verification of the *PMS2* variant c.2275+1G>C long range PCR (LR-PCR) was performed according to the instructions provided in the LR-PCR Kit (TaKaRa, Tokyo, Japan) using primers and conditions as previously described (Vaughn et al., 2010). A second set of primers was used for nested PCR avoiding polymorphisms located in the primer sequences. The primer sequences were tested using SNPCheck^14^ and are available upon request.

### Immunohistochemical Analysis

Formalin fixed, paraffin-embedded samples of CRC or adenomas were selected for immunohistochemical studies. Immunohistochemical evaluation, on 3 μm thick sections, was done using the following Ready-to-use antibodies MLH1 (clone ES05), PMS2 (clone EP51, MSH2 (clone FE11) and MSH6 (EP49) from Agilent following the manufacturer’s instructions. The staining took place on the Omnis from Agilent utilizing the EnVision Flex + detection kit (GV800). The sections were counterstained with hematoxylin.

Some samples have been analyzed in other departments of pathology, and historically only MLH1 and MSH2 or MLH1, MSH2, and MSH6 were analyzed. These samples might have been analyzed using other kits, however, all samples have been analyzed as part of a clinical evaluation by experienced pathologists.

#### Interpretation of IHC Stainings

The IHC stainings were interpreted by trained colorectal pathologists as either positive (retained nuclear staining in any number of tumor cells) or negative (complete loss of nuclear staining in all tumor cells). Normal colonic crypt epithelium adjacent to the tumor, lymphoid cells and stromal cells served as internal positive controls. In addition, on slide positive controls are a routine practice in our IHC laboratory.

## Results

**FIGURE 2:**
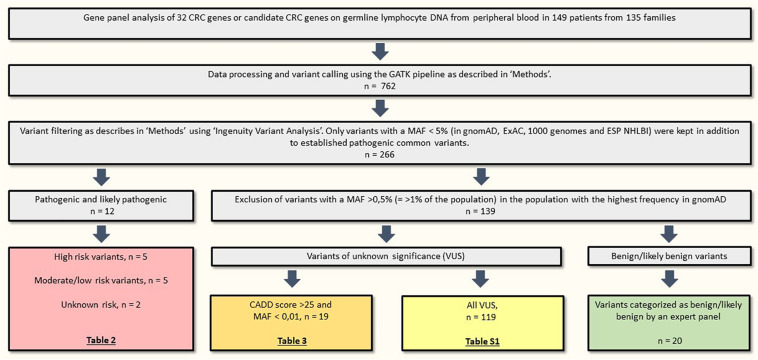
Variant filtering. CRC, colorectal cancer; MAF, minor allele frequency; n, number of variants.

We identified 12 pathogenic or likely pathogenic variants in 15 patients (10.1% of the included patients), listed in Table 2. High risk variants in *MSH2*, *POLE* and *APC* were identified in 3/85 families with CRC (3.5%) and variants in *PMS2* were identified in 2/50 patients (4%) from the early onset CRC cohort. A total of 119 variants of unknown significance (VUS) were detected and are listed in Supplementary Table S1; the 19 most interesting VUS, based on frequency and CADD scores, are listed in Table 3. Variant filtering is summarized in Figure 2.

**TABLE 2:** Pathogenic and likely pathogenic variants.

**Family ID (pt.ID)**	**Gene**	**Transcript variant**	**Protein variant**	**Cancer localization and adenomas (age)**	**Family history^1^**
**High risk variants**					
14 (14;16 and 14;20)	*APC*	c.289G>A	p.(Gly97Arg)	14;16: Duodenal cancer (78), IHC = n/a. >100 colonic adenomas. 14;20: Transverse colon cancer (46), IHC = n/a. 25 colonic adenomas.	Sister A^+^: Four primary colon cancers (57), <10 colonic adenomas and a hepatic mucinous cystadenocarcinoma (69). Also had Caroli disease. Sister B^?^: Breast cancer (55). Mother^?^: Colon cancer (66). Mat. 2DR^?^: Testicular cancer (68).
165 (165;10)	*MSH2*	c.2168C>T	p.(Ser723Phe)	Ascending colon cancer (47), IHC = MSH2 absent. Cancer in the major duodenal papilla (51), IHC = MLH1/PMS2 absent (MLH1 promoter methylation analysis showed methylation). 2 advanced adenomas.	Daughter^+^: Rectal cancer (25), 2 advanced, colonic adenomas. Mother^–^: Transverse colon cancer (44) and 1 advanced, colonic adenoma. Mat. 2/3DR^–^: Several cases of CRC (58–80).
309 (309;10)	*PMS2*	c.736_741delins TGTGTGTGAAG	p.(Pro246Cysfs*3)	Cecal cancer (29), IHC = PMS2 absent.	Mother^?^: Breast cancer (68), endometrial cancer (73) and one advanced TA. Pat. 2DR^?^: Cervical cancer (50)
409 (409;10)	*PMS2*	2275+1G>C	p.(?)	Ascending colon cancer (36), IHC = PMS2 absent.	No cancers in first degree relatives.
27 (27;10)	*POLE*	c.1089C>A	p.(Asn363Lys)	Ascending colon cancer (28), IHC = Intact. Synchronous colorectal cancer (40).	Mother^–^: Malignant melanoma (68) and lung cancer (84). Father^–^: Colon cancer (67). Pat. 2DR^–^: Sigmoid colon cancer (50)
**Moderate or low risk variants**					
6(6;142)	*APC*	c.3920T>A	p.(Ile1307Lys)	Sigmoid colon cancer (47), IHC = Intact.	Mother^?^: Died <40 years old (non-malignant disease) Father^?^: Not CRC. Pat. 2-4DR: Four CRCs (61–81)^?^; Sigmoid colon cancer (58) ^–^; Synchronous ovarian cancer and colon cancer (50)^?^
12 (12;82)	*CHEK2*	c.1100delC	p.(Thr367Metfs15*)	Transverse colon cancer (66), IHC = MLH1/PMS2 absent (MLH1 promoter methylation analysis showed methylation).	Son^?^: Rectal cancer (42). Siblings^?^: Sigmoid colon cancer (46) and lung cancer (50); 3 advanced adenomas.
200 (200;10)	*CHEK2*	c.1100delC	p.(Thr367Metfs15*)	Colon cancer (36), IHC = MSH6 absent.	Not CRC in first degree relatives.
55 (55;8)	*EXO1*	c.2212-1G>C	p.(?)	Sigmoid colon (65), IHC = Intact.	Daughter^+^: Descending colon cancer (36). Father^?^: Sigmoid colon (76).
397 (397;10)	*EXO1* *MUTYH*	c.2212-1G>C c.536A>G	p.(?) p.(Tyr179Cys)	Rectal cancer (31), IHC = n/a.	Parents^?^: Not CRC. Pat. 2DR^?^: Leukemia (64).
112 (112;80)	*GALNT12*	c.907G>A	p.(Asp303Asn)	Rectal cancer (57), IHC = Intact.	Father^?^: Colon cancer (66). Brother^?^: Colon sigmoid cancer (50). Pat. 2DRs^?^: Prostate cancer (78), melanoma (83), rectal cancer (84) and 7 adenomas; Ovarian cancer (76) and bladder cancer 79.
329 (329;10)	*MUTYH*	c.536A>G	p.(Tyr179Cys)	Ascending colon cancer (33), IHC = Intact.	No cancers in first- or second-degree relatives.
**Variants with unknown risk (monoallelic pathogenic variants)**					
84 (84;14)	*MSH3*	c.2319-1G>A	p.(?)	Sigmoid colon cancer (47), IHC = n/a.	Father^?^: Rectal cancer (76). Pat. 2-4DR^?^: Rectal cancer (58) and appendix cancer (58); Rectal cancer (55) and lung cancer (62); Rectal cancer (54); Breast cancer (49).
136 (136;12)	*NTHL1*	c.268C>T	p.(Gln90*)	Sigmoid colon cancer (49), IHC = Intact. Basal cell carcinoma (51), two adenomas.	Father^?^: Colon cancer (81). Pat. 2DRs^?^: Breast cancer (62); Breast cancer (67); Colon cancer (61) and 3 adenomas Mat. 2DR^?^: Breast (71).

*^1^Family history includes verified cases of cancers and advanced colonic adenomas (villous morphology, size > 10 mm. or high-grade dysplasia) in first- and second-degree relatives. Single cases of basal cell skin cancer are not listed. Family members are separated by semicolons. TA, tubular adenoma; LG, low grade dysplasia; Mat, maternal; Pat., paternal; 2DR/3DR/4DR, second/third/fourth-degree relatives. Carrier status: ^?^ Variant status unknown, ^+^ carrier, ^–^ non-carrier. IHC, immunohistochemical analysis; n/a, not available.*

**TABLE 3:** Selected variants of unknown significance.

**Family**	**Gene**	**Transcript variant**	**Protein variant**	**CADD score**	Impact on splicing*	**Highest freq. (population)**^†^	**Allele Count (homo.)^#^**	**Clinvar^**	**Comment**
408	*APC*	c.2026A>G	p.(Ile676Val)	25.200	No	0.0000353 (NFE)	6 (0)	C3 (5)	*Did not have polyposis*
338	*AXIN2*	c.1975C>T	p.(Arg659Trp)	33.000	No	0.0006547 (SAS)	66 (0)	C2 (1), C3 (2)	
20	*BLM*	c.2389G>A	p.(Ala797Thr)	32.000	No	–	–		
67	*BUB1*	c.1457A>G	p.(Asp486Gly)	26.900	No	–	–		
162	*CDH1*	c.2335C>T	p.(Arg779Trp)	33.000	No	–	–	C3 (5)	
379	*CDH1*	c.2474C>T	p.(Pro825Leu)	25.900	No	0.0000289 (AMR)	4 (0)	C3 (5)	
69	*CHEK2*	c.497A>C	p.(Asn166Thr)	25.900	No	–	–		
110	*CHEK2*	c.1427C>T	p.(Thr476Met)	25.600	No	0.0005424 (NFE)	77 (0)	C3 (8), C4 (10)	
7	*FOCAD*	c.3086A>T	p.(Tyr1029Phe)	28.700	No	0.0000088 (NFE)	1 (0)		*Does not segregate in the family*
8	*FOCAD*	c.5376A>T	p.(Lys1792Asn)	26.400	No	0.0005643 (NFE)	67 (0)		*Does not segregate in the family*
406	*GALNT12*	c.303C>G	p.(His101Gln)	25.300	No	0.0003835 (NFE)	23 (0)	C3 (2)	
309	*MLH3*	c.1234A>G	p.(Lys412Glu)	25.400	No	0.0004564 (NFE)	110 (0)	C2 (1)	*Also has a pathogenic PMS2 variant*
366	*MLH3*	c.3533C>T	p.(Pro1178Leu)	27.000	No	0.0000264 (NFE)	3 (0)		
344	*POLD1*	c.961G>A	p.(Gly321Ser)	25.400	No	0.0007252 (NFE)	90 (0)	C3 (9)	
113	*POLE*	c.797G>A	p.(Arg266Gln)	26.000	No	0.0000088 (NFE)	1 (0)	C3 (2)	*Located just outside the END^$^*
92	*RPS20*	c.98A>T	p.(Glu33Val)	26.400	CDS	–	–		*Segregates with CRC in the family*
350	*SMAD9*	c.1161C>A	p.(Asn387Lys)	25.200	No	0.0002287 (NFE)	27 (0)		
58	*SMAD9*	c.1171G>A	p.(Ala391Thr)	25.200	No	0.0001231 (AFR)	9 (0)		
121	*TP53*	c.814G>A	p.(Val272Met)	27.200	No	0.0000088 (NFE)	1 (0)		*Hematopoietic clone*

Variants with a CADD score > 25 and an allele frequency < 0,001 in the population with the highest alternate allele frequency in exomes in gnomAD are listed. *BRCA1*, *BRCA2*, *CDH1*, MMR-, and *PTEN* variants categorized as benign or likely benign by expert panels (such as Enigma or InSiGHT) have been discarded. * Impact on splicing was evaluated using Alamut. Only differences in canonical splice site strength >10% (based on MaxEntScan) was noticed. As for cryptic splice sites, these are only mentioned if at least 4/5 *in silico* programs indicated that a new splice site was created. CDS, cryptic donor site. ^†^The highest allele frequency in exomes in gnomAD in any population. NFE, Non-Finnish European; SAS, South Asian; AMR, Latino; AFR, African. ^#^Allele count: Number of alleles with the variant found in exomes in gnomAD. The number in () refers to the number of homozygous individuals. ^∧^Clinvar: C2: Likely benign, C3: Uncertain significance, C4: Likely pathogenic. Number in () refers to the number of classifications. *^$^*Exonuclease domain.

### Pathogenic and Likely Pathogenic High-Risk Variants

The missense variant c.289G>A, p.(Gly97Arg) in *APC* was identified in two siblings (no. #14;16 and #14;20) with attenuated familial adenomatous polyposis (AFAP) phenotype. Segregation analysis revealed that a third sibling with AFAP also carried the variant. The variant has previously been reported in a Chinese patient with mild FAP (Wang et al., 2019). The variant creates a cryptic acceptor splice site and interrupts normal splicing; this family and the results of the functional analyses have already been published (Djursby et al., 2020). Based on these data, we consider the APC c.289G>A variant likely pathogenic.

**FIGURE 3:**
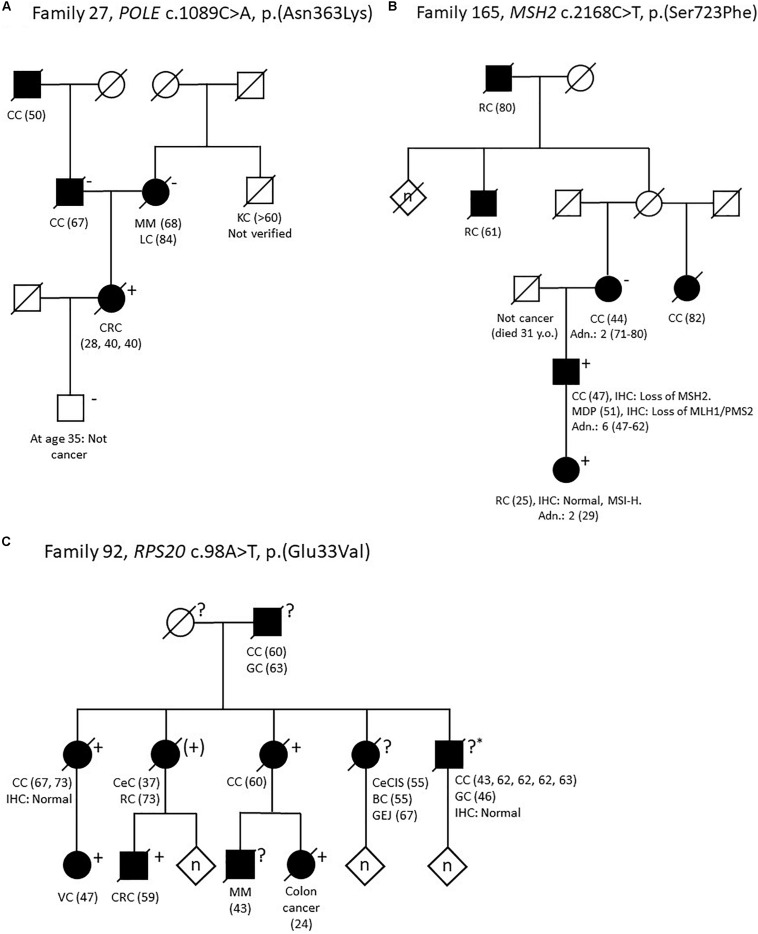
Pedigrees of selected families. **(A)** Family 27. *POLE* c.1089C>A, p.(Asn363Lys). **(B)** Family 165. MSH2 c.2168C>T, p.(Ser723Phe). **(C)** Family 92. RPS20 c.98A>T, p.(Glu33Val). Adn., colonic adenomas; BC, breast cancer; CC, colon cancer; CeC, cervical cancer; CeCIS, cervical carcinoma *in situ*; CRC, colorectal cancer; GC, gastric cancer; GEJ, malignant gastroesophageal junction tumor; IHC, immunohistochemistry; KC, kidney cancer; LC, lung cancer; MDP, malignant major duodenal papilla tumor; MM, malignant melanoma; MSI-H, microsatellite instability high; RC, rectal cancer; VC, vulvar cancer; Y.o, years old. Numbers in parentheses refer to age at diagnosis. + variant carrier, (+) obliged variant carrier (not tested), ? unknown carrier status, ?* unknown carrier status (analysis failed), −non-carrier.

One patient (no. #27;10) had a likely pathogenic variant in *POLE*: c.1089C>A, p.(Asn363Lys). This variant is not reported in population allele frequency databases, but has been identified in two large families with multi tumor phenotypes (Rohlin et al., 2014; Vande Perre et al., 2019). The variant affects the highly conserved amino acid Asn-363 in the exonuclease domain of *POLE*, and so far only missense variants in this domain have been confirmed pathogenic (Bellido et al., 2016). Segregation analysis in the parents on healthy FFPE tissue indicates that the variant was *de novo* in our patient, which considerably increases the pathogenicity of the variant. Family data can be found in Figure 3, pedigree A. Based on *in silico* data, our clinical data and co-segregation data in the two large published families we classify this variant as likely pathogenic.

We identified two *PMS2* variants in two patients with early onset CRC (no. #309;10 and no. #409;10). The first *PMS2* variant was an indel: c.736_741delinsTGTGTGTGAAG, p.(Pro246Cysfs^∗^3). It is categorized as pathogenic by InSiGHT and has been identified in several Danish patients (Okkels et al., 2019). The second *PMS2* variant was a splice site variant c.2275+1G>C and has to our knowledge not previously been reported. To avoid analysis of the *PMS2* pseudogenes, the variant was confirmed using LR-PCR. Immunohistochemical analysis (IHC) also showed loss of the PMS2 protein in the tumor. Since the patient is deceased, it has not been possible to perform mRNA analyses, but the variant is predicted to disrupt normal splicing completely by five out of five *in silico* splicing programs in Alamut, and we consider it to be likely pathogenic.

In family 165 (Figure 3, pedigree B) we identified the *MSH2* variant c.2168C>T, p.(Ser723Phe). The variant had already been detected in the family (Nilbert et al., 2009), but was considered to be a variant of unknown significance (which is also supported by InSiGHT classification) and the family was included in this study to search for alternative explanations. The index person (no. #165;10), who carries the *MSH2* c.2168C>T variant, have had two primary cancers: Colon cancer (IHC: lack of MSH2 expression, microsatellite instability (MSI) status unknown) and an adenocarcinoma at the major duodenal papilla (IHC: lack of MLH1/PMS2, and methylation of the *MLH1* promoter). His daughter had rectal cancer 25-years-old [IHC: normal, but MSI-high (MSI-H)] and she also carries the *MSH2* variant. His mother developed colon cancer 44 years-old-old (IHC and MSI status unavailable), but she does not carry the variant. However, the family history is complex as the mother is also predisposed to CRC from another branch of the family, and the father of the index person died only 31-years-old of non-malignant disease. Ser-723 is a highly conserved amino acid and the variant is predicted to be disease causing by several *in silico* prediction tools. Several groups have evaluated this variant using *in vitro* MMR activity assays, yeast assays or murine or human embryonic stem cells; all studies indicate that the variant interrupts normal mismatch repair function and is pathogenic (Gammie et al., 2007; Drost et al., 2012; Houlleberghs et al., 2016; Rath et al., 2019). Based on a suggested functional effect in four studies, in combination with our data with co-segregation in two individuals with early onset CRC, we consider the *MSH2* c.2168C>T variant likely pathogenic.

### Pathogenic and Likely Pathogenic Moderate and Low Risk Variants

Two patients (no. #329;10 and #397;10) were heterozygous for the well-established, pathogenic variant in *MUTYH* c.536A>G, p.(Tyr179Cys). In *APC* we detected the frequent c.3920T>A, p.(Ile1307Lys) variant in a woman with colon cancer at age 47 years (no. #6;142). In *CHEK2* we detected the pathogenic frameshift c.1100del, p.(Thr367Metfs^∗^15) variant in two patients (no. #12;82 and #200;10). We identified the *GALNT12* variant c.907G>A, p.(Asp303Asn) in one person (no. #112;80). This variant has been described several times, and although the role of *GALNT12* in familial cancer is controversial, this particular variant most likely confers a moderate risk (Guda et al., 2009; Clarke et al., 2012; Seguí et al., 2014; Lorca et al., 2017; Evans et al., 2018). In *EXO1* we identified the c.2212-1G>C variant in two individuals (no. #55;8 and #397;10). This variant appears to confer only a low risk (Jagmohan-Changur et al., 2003; Talseth-Palmer et al., 2016).

### Variants of Unknown Significance

In family 92, a large Amsterdam I positive family with 13 cases of CRC (IHC analysis in two tumors from two different patients showed normal expression of the MMR-proteins) in addition to other cancers, we identified the *RPS20* variant c.98A>T, p.(Glu33Val). This variant is absent from all population allele frequency databases and has to our knowledge not previously been reported in the literature. Glu-33 is a highly conserved amino acid and the variant is predicted to be pathogenic by several *in silico* programs and also to affect splicing by four out of five *in silico* splicing programs in Alamut by creating a new cryptic donor site, which may lead to a frameshift due to a loss of seven base pairs. Segregation analysis showed that five relatives with CRC from age 24 to 73 years old also carried the variant (Figure 3, pedigree C).

In one person (#121;10) we identified the likely pathogenic *TP53* variant c.814G>A, p.(Val272Met). The family history consists of three cases of CRC, two hematological cancers and one case of prostate cancer; all cancers were diagnosed after age 50 years. The variant had a VAF of 24% in two separate blood samples suggesting that the variant was either a hematopoietic clone or a case of classic mosaicism (the patient had the tumor removed surgically >10 years ago, which almost certainly excludes circulating tumor DNA from this tumor as a possibility). In order to clarify this issue, we sequenced tumor tissue and healthy non-malignant tissue; the variant was found in 8% of the reads in the tumor and was not found in healthy tissue indicating that the clone most likely represents a hematopoietic clone with lymphocyte infiltration in the tumor.

We identified six VUS in the MMR genes and eleven VUS in *APC* (listed in Supplementary Table S1), of which two variants – based on allele frequency – are particularly interesting: *MSH6* c.3232G>C, p.(Val1078Leu) and *APC* c.4318C>T, p.(Pro1440Ser). The *MSH6* c.3232G>C variant was detected in a patient with CRC from the early onset cohort (no. #329;10), who also had a pathogenic monoallelic *MUTYH* variant. The tumor was microsatellite stable and had normal MSH6 expression, and along with benign *in silico* prediction, the variant has a low probability of being pathogenic. The *APC* c.4318C>T variant has not previously been reported and affects a highly conserved amino acid. The patient (no #1;52) had colon cancer 52-years-old in addition to three small tubular adenomas. He had two brothers with childhood leukemia and adult-onset serrated polyposis syndrome, respectively, and is predisposed to CRC on the paternal side (however, the variant does not segregate with CRC in a 4th degree paternal relative with CRC 49-years-old and nine adenomas). According to the family, the maternal family history is positive with two cases of brain tumors in addition to a verified case of follicular thyroid carcinoma. Unfortunately, no functional data on the variant are available.

In one patient (no. #106;10) we identified the *BUB1* variant c.1321A>G, p.(Thr441Ala). The variant is predicted to introduce a cryptic acceptor splice site 45 base pairs into exon 12 and could lead to an in-frame loss of 15 amino acids in the protein. *BUB1* variants have in addition to colon cancer been associated with mosaic variegated aneuploidy syndrome (MVAS), due to *BUB1*’s role as a component of the spindle assembly checkpoint, and dysmorphic features. Data from our patient concerning dysmorphic features were not available, and the variant does not segregate with CRC in a 4th degree relative with CRC at age 48.

In *FAN1*, we identified the frameshift variant c.922_923del, p.(Val308Cysfs^∗^5), in a person with metachronous CRC (no. #143;8), who was a second degree relative in an Amsterdam I positive family. The variant is reported in exomes in gnomAD with an allele frequency of 0.023% in non-Finnish Europeans and has been classified as likely pathogenic in ClinVar. However, the variant does not segregate with another case of rectal cancer at age 49 or a case of cancer with unknown origin at age 59 in the family.

In addition, we identified several missense variants in *CHEK2* and one in *POLD1* that might have a moderate impact on CRC risk (Table 3).

### Monoallelic Pathogenic Variants in Genes With Autosomal Recessive Inheritance

We detected the previously reported *NTHL1* nonsense variant c.268C>T, p.Gln90^∗^ in one patient (no. #136;12). To our knowledge, the significance of monoallelic, pathogenic *NTHL1* variants is currently unknown (Weren et al., 2015). Another patient (no. #84;14) was heterozygous for the likely pathogenic *MSH3* variant c.2319-1G>A (Adam et al., 2016). The patient also carried a second *MSH3* intron variant, c.2436-13G>T not predicted to affect splicing by *in silico* analysis (Alamut). Due to the method (short read sequencing) used in this study, it was not possible to unravel if the variants are in *cis* or *trans*. Since *MSH3*-related CRC is inherited in an autosomal recessive pattern, the variants cannot explain the apparently dominant inheritance pattern in the family.

## Discussion

In this study, we performed gene panel analysis of 32 CRC associated genes in two cohorts of patients: (1). Patients with early onset CRC (*n* = 50) and (2). Patients from families with familial CRC (*n* = 99 patients from 85 families). The great majority of patients had MMR-proficient tumors, based on IHC analysis, and all patients (*n* = 7) from the familial CRC cohort with abnormal IHC expression had a relative with normal IHC analysis. This was consistent with the results of previous genetic testing of the MMR genes where ∼90% of the participants, or an affected relative, had had MMR analysis performed without identification of a pathogenic or likely pathogenic variant (Table 1).

In the cohort with familial CRC we identified three high-risk pathogenic variants in *APC*, *MSH2* and *POLE*. The *MSH2* variant, c.2168C>T, p.(Ser723Phe) had previously been identified, but since new knowledge have emerged, the variant was reclassified to a likely pathogenic variant from a VUS. The likely pathogenic *APC* variant c.289G>A, p.(Gly97Arg) was – for reasons unknown – not identified or interpreted as pathogenic when *APC* analysis was performed 20 years ago, but since the family fulfils *APC*-testing criteria the variant would normally have been detected through routine genetic handling. The *APC*-screening was originally performed using only Sanger sequencing, and these two cases emphasize the importance of a regular reassessment of genetically unresolved families with apparently inherited cancer with either a reassessment of variants of unknown significance or repeated NGS-based analyses. The *POLE* c.1089C>A, p.(Asn363Lys) variant was found in an Amsterdam I positive family, where two affected persons turned out to be phenocopies. They had milder phenotypes with only one tumor at a higher age (67 and 50 years old, respectively) compared to three syn- and metachronous CRCs at age 28 and 40 in the index patient. This case clearly illustrates the great importance of choosing the most severely affected family member for genetic analysis.

Thus, although we identified pathogenic variants in 8 families out 85 families (9.4%) only three variants were clinically actionable and two variants were, or should have been, detected previously.

In the early onset cohort, we identified two pathogenic/likely pathogenic *PMS2* variants in addition to several low or moderate risk variant including a pathogenic *MUTYH* variant, identified in two unrelated CRC patients. The clinical impact of pathogenic *PMS2* variants are currently debated, but it is widely accepted that *PMS2* variants confer a much lower cancer risk than the other MMR genes. For now, however, the families are being handled as classic Lynch Syndrome families. Monoallelic *MUTYH* variants are associated with a ∼two-fold risk of developing CRC, and in Denmark carriers of monoallelic pathogenic *MUTYH* variants, who have a first-degree relative with CRC, are offered colonoscopy surveillance every 5 years. Thus, the diagnostic yield of clinically actionable variants in the early onset cohort were 8% (4 out of 50 patients).

The genetic background of CRC for a large proportion of the patients in our study is still unresolved, which can have several explanations.

Firstly, our gene panel consisted of only CRC-related genes, and we might have had a higher diagnostic yield if our gene panel had included more known cancer predisposition genes.

Secondly, we did not include copy number variation (CNV) analysis in our study. CNVs have been estimated to account for up to 10% of all pathogenic variants (with great differences from gene to gene) suggesting that we might have missed et least one or a few CNVs in our cohort (LaDuca et al., 2019). Clinical follow up on the patients revealed that one patient actually had been diagnosed with juvenile polyposis syndrome due to a deletion of exon 9 to 10 in *SMAD4.* She developed colon cancer at age 36 and had had three colonic adenomas removed (one with high grade dysplasia). After the diagnosis she had another polyp removed which was interpreted as either an inflammatory or a juvenile polyp. In total, about one out of three patients had had CNV analysis performed in a clinical setting, but no further CNVs had been detected (unpublished data). The *SMAD4* case also exemplifies the overlapping features of CRC-related syndromes: The patient was highly suspicious of having HNPCC/Lynch Syndrome but ended up with a diagnosis of juvenile polyposis.

Thirdly, some patients probably have pathogenic variants in regions that our analysis was not designed to capture, such as deep intronic or regulatory variants in known cancer genes or variants in genes not yet identified or associated with CRC. Many genes have been suggested as candidate colorectal genes, and especially genes such as *POLE2*, *MRE11*, and *POT1* appear to be interesting genes as well as epigenetic changes in *PTPRJ* (Venkatachalam et al., 2010; Chubb et al., 2016; Terradas et al., 2020). Another possibility is a non-mendelian predisposition to CRC. Polygenic inheritance has been shown to explain up to ∼15% of the familial CRC risk (Frampton et al., 2016) and at least two cases with CRC and possible digenic and oligogenic inheritance have been reported; these patients had variants in *MUTYH* and *OGG1* (both involved in the base excision repair pathway) and in *APC*, *OGG1*, *EXO1* and *POLQ*, respectively (Morak et al., 2011; Ciavarella et al., 2018). *OGG1* was not part of our gene panel, and its role in hereditary CRC/polyposis is controversial (Smith et al., 2013; Mur et al., 2018). Intriguingly, we identified the same *EXO1* variant in a patient with rectal cancer 31-years-old (no. 397;10) from the early onset CRC cohort who also carried a monoallelic, pathogenic *MUTYH* variant. Although *EXO1* (mainly involved in mismatch repair) and *MUTYH* (mainly involved in base excision repair) are not involved in the same pathway, they are both involved in DNA repair pathways. In order to reveal if she had other low or moderate risk variants – and thus could represent a case of oligogenic inheritance – we plan to perform whole genome sequencing (WGS) as next step.

As expected, we detected a high number of VUS, and some may – as more data become available – be reclassified as either class 1/2 or 4/5. An example of a VUS with a high potential of being reclassified to (likely) pathogenic is the *RPS20*, c.98A>T, p.(Glu33Val), variant. Only two families, one large family with multiple cases of CRC and a truncating *RPS20* variant (c.147dupA, p.Val50SerfsTer23), and one small family with a splice site variant shown to disturb normal splicing (c.177+1G>A), have been reported in addition to two individuals with early onset CRC and without published family cancer history (Nieminen et al., 2014; Broderick et al., 2017; Thompson et al., 2020). The latter individuals had a missense variant, p.Val54Leu, and a frameshift variant, p.Leu61GlufsTer11, respectively. All analyzed tumors, including those in our family, have shown MMR-proficient tumor phenotype based on IHC analysis. *RPS20* encodes a ribosomal protein which is a part of the S40 subunit. *RPS20* has been suggested to be involved in cell proliferation and regulation, and as a stabilizer of p53 (Nieminen et al., 2014) but recently Krishnan et al. (2018) provided evidence of a critical interaction between *RPS20* and *GNL1* – a nucleolar ATPase also involved in cell cycle regulation – which regulates and promotes the G1/S phase, and thus provided documentation of a possible link to tumorigenesis. Several factors support that the c.98A>T variant is pathogenic: Strong segregation analysis in the family presented in our study, *in silico* splicing prediction and the fact that the variant has not previously been reported or cataloged in gnomAD. *RPS20* is a very promising colorectal candidate gene, and the identification of three families with variants segregating with disease is strong evidence. However, since the role of *RPS20* in CRC is not yet fully established, we categorize the variant as a VUS. Three out of four of the previously published variants in *RPS20* were located in exon 3 or close to exon 3/4 boundaries. These variants have only been associated with CRC. The variant identified in the family presented in our study were located in exon 2 (close to the exon 2/intron 2 junction), and this family has a pedigree with 12 cases of CRC, but also other cancer types such as early onset vulva cancer, melanoma, breast cancer and esophageal/gastric cancer (Figure 2, pedigree C). Our family also has the by far youngest affected *RPS20* carrier, a female who was diagnosed with CRC 24-years-old. The other families also had early onset CRC cases at age 38, 39, and 41 respectively. Nieminen et al. (2014) did not provide detailed data on age at onset, but the mean age at onset of CRC were 52.3 years. Due to the scarcity of families with *RPS20* variants, data on genotype-phenotype correlations are premature, but if the c.98A>T variant turns out to be pathogenic it will not only confirm the role of *RPS20* in hereditary CRC, but it will also expand the phenotypic spectrum of *RPS20* related cancer significantly. Due to the very high probability of *RPS20* truly being a new cancer gene, we recommend inclusion of *RPS20* in cancer gene panels.

An example of a complicated VUS is the *TP53* c.814G>A variant. The family does not meet *TP53* testing criteria [i.e., the 2015 version of the Chompret Criteria (Bougeard et al., 2015)] and the variant was solely identified because our multigene panel analysis was performed. *TP53* variants with a reduced mutant to wild-type allele ratio (MWTAR), for example <25% or 30%, are a common issue when analyzing multi gene panels as discussed by Weitzel et al. (2018); their study also showed that *TP53* variants with reduced MWTAR were more likely to represent hematopoietic clones when identified in multigene panels and in older patients, which was also the case in our family.

We detected several other pathogenic and interesting VUS in moderate penetrance genes such as *CHEK2* and *GALNT12*, however, until we have reached a better understanding of the consequences of combinations of low- and moderate risk variants, we consider genetic testing of these genes in a clinical setting premature.

We identified only one variant in *FAN1* with an allele frequency of <0.1%, namely the c.922_923del variant. The variant has been published in two patients with a suspected genetic predisposition to cancer but neither first- or second- degree-relatives were affected by CRC (Fievet et al., 2019). *FAN1* was proposed to be a CRC candidate gene in 2015 (Seguí et al., 2015), but the evidence of its role in hereditary CRC is still very limited (Broderick et al., 2017; Fievet et al., 2019). Our data further questions the role of *FAN1* in hereditary cancer, and like Fievet et al. (2019), we also suggest excluding this gene from cancer gene panels.

In general, our study – a retrospective cohort study consisting primarily of highly selected MMR-proficient individuals – showed the highest diagnostic hit rate in families with a high burden of adenomas or with an exceptionally early age at onset. The youngest variant carriers with CRC in the families with (possible) high-risk variants were diagnosed with CRC at a mean of 28.4 years (age 28 (*POLE*, c. 1089C>A), 25 (*MSH2*, c.2168C>T), 29 (*PMS2*, c.736_741delinsTGTGTGTGAAG), 36 (*PMS2*, c.2275+1G>C) and 24 (*RPS20*, c.98A>T) compared to an average age of 40.5 years of the youngest person diagnosed with CRC in the rest of the participant’s families (only 1st to 3rd degree relatives are included). In total, only eight families had a person with CRC before age 30, and we found a (possible) genetic explanation in 50%. Thus, when reevaluating families/patients with previous MMR-analysis and without polyposis, our data suggest that a limited number of genes – such as *APC* (primarily in order to identify AFAP families) and *MUTYH* (due to the high population carrier frequency) – is sufficient to capture the majority of families with a hereditary cancer predisposition syndrome. The exception appears to be when very early onset cases of CRC have occurred, and also if the personal history or family history suggests a syndromic etiology such as Peutz-Jeghers syndrome, juvenile polyposis syndrome, Cowden syndrome etc. The age limit of very early onset CRC is arbitrary, but a proposal could be age 40. These families would benefit from a larger gene panel analysis.

Since most patients referred for genetic evaluation have not been genetic tested previously, the approach in these families needs to be different. In Denmark – with a public health care system allowing all citizens access to relevant health care regardless of income/insurance – both cohorts are eligible for genetic counseling and -testing and the majority are offered analysis of a clinical CRC gene panel consisting of 17 CRC and polyposis related genes (as described in the section “Introduction”). The Collaborative Group of the Americas on Inherited Gastrointestinal Cancer recently published a recommendation regarding gene panel testing in hereditary CRC or polyposis, and they recommend that multigene testing as a minimum includes the MMR-genes, *EPCAM*, *APC*, *MUTYH*, *BMPR1A*, *SMAD4*, *PTEN* and *STK11* (Heald et al., 2020). Although some of the genes are primarily relevant in case of polyposis (*PMPR1A*, *SMAD4*, *PTEN*, *STK11*), implementation of this gene panel would probably be the approach with lowest costs. Another approach is testing of one large cancer gene panel irrespective of phenotype. Studies analyzing very large cancer gene panels, not based on phenotype, have widened the phenotypic spectra for a number of cancer genes (Espenschied et al., 2017; Rohlin et al., 2017; LaDuca et al., 2019) and this approach would also catch rare causes of CRC. Although large gene panels generate more VUS that can be challenging to interpret and handle clinically, they are more efficient in terms of price and time. A third and fourth approach is targeted WES or WGS, respectively. WGS has the advantage of generating the greatest amount of data including (i) promoter, regulatory and deep intronic variants, which is very relevant to look for in established cancer genes (ii) reliable CNV data, (iii) data on structural rearrangements (iv) data on all SNPs making it possible to calculate polygenic risk scores, and (v) readily available data on new genes/variants when new knowledge comes forth. On the other hand, WGS is the most expensive and time-consuming analysis (when it comes to variant interpretation). Costs of WGS are currently approaching those of WES, but both are high-cost approaches, and the gray zone between clinical evaluation and research, as well as the ethical dilemmas of performing large genomic analyses, are issues which should be discussed. A paradigm shift from phenotype-based cancer gene panels to larger genomic analyses seems inevitable though.

The combination of young age at onset and a lack of CRC in the family history would suggest recessive inheritance or high-risk *de novo* variants, and the complete lack of other high-risk variants than the *PMS2* variants in our early onset CRC cohort was unexpected. In the familial cancer cohorts, a number of the families are indeed highly suspicious of having a genetic cancer syndrome, and although novel cancer genes probably only account for a very small percentage of cases (Chubb et al., 2016) further studies are warranted in order to elucidate the genetic background of hereditary cancer and to identify novel cancer genes. In order to search for other explanations in our cohorts, selected individuals are now undergoing WGS, which will hopefully help to clarify the disease-causing mechanisms in a larger proportion of the individuals.

## Data Availability Statement

The datasets for this article are not publicly available due to the data being the property of a third party. All class 3, 4, or 5 variants have been uploaded in the article, and relevant variants will also be uploaded in ClinVar. Requests to access the datasets should be directed to the corresponding author, and certain parts can be shared upon reasonable request.

## Ethics Statement

The studies involving human participants were reviewed and approved by The Danish Committee on Health Research Ethics, reference: H-4-2014-050. The patients/participants provided their written informed consent to participate in this study. Written informed consent was obtained from the individual(s) for the publication of any potentially identifiable images or data included in this article.

## Author Contributions

MD, KW, TO, A-MG, CT, and MN initiated and designed the project. MD included the great majority of the patients and wrote the first draft of the manuscript together with KW, A-MG, and TO. MM, LB, HO, GW, JH, and FW contributed to the method section. MM and LB were responsible of the laboratory analyses, and interpreted data together with MD, KW, TO, JF, and A-MG. HO and FW also contributed to the laboratory analyses and interpretation of data. The RPS20 family was clinically handled by A-BS. GW and JH performed the pathological analyses and reviewed tissue samples. All authors read the manuscript critically and approved the final version of the manuscript.

## Conflict of Interest

The authors declare that the research was conducted in the absence of any commercial or financial relationships that could be construed as a potential conflict of interest.

## Footnotes

1 http://ingenuity.com

2 http://www.1000genomes.org

3 http://exac.broadinstitute.org

4 http://gnomad.broadinstitute.org

5 http://evs.gs.washington.edu/EVS

6 http://enigma.ini.usc.edu/

7 https://insight-database.org/

8 https://www.interactive-biosoftware.com

9 https://www.lovd.nl/

10 https://www.ncbi.nlm.nih.gov/clinvar/

11 https://cancer.sanger.ac.uk/cosmic

12 https://pubmed.ncbi.nlm.nih.gov/

13 https://software.broadinstitute.org

14 https://secure.ngrl.org.uk/SNPCheck/

## References

[B1] Adam R. Spier I. Zhao B. Kloth M. Marquez J. Hinrichsen I. (2016). Exome sequencing identifies biallelic Msh3 germline mutations as a recessive subtype of colorectal adenomatous polyposis. *Am. J. Hum. Genet.* 99 337–351. 10.1016/j.ajhg.2016.06.015 27476653 PMC4974087

[B2] Bellido F. Pineda M. Aiza G. Valdés-Mas R. Navarro M. Puente D. A. (2016). POLE and POLD1 mutations in 529 kindred with familial colorectal cancer and/or polyposis: review of reported cases and recommendations for genetic testing and surveillance. *Genet. Med.* 18 325–332. 10.1038/gim.2015.75 26133394 PMC4823640

[B3] Bougeard G. Renaux-Petel M. Flaman J. M. Charbonnier C. Fermey P. Belotti M. (2015). Revisiting Li-Fraumeni syndrome from TP53 mutation carriers. *J. Clin. Oncol.* 33 2345–2352. 26014290 10.1200/JCO.2014.59.5728

[B4] Broderick P. Dobbins S. E. Chubb D. Kinnersley B. Dunlop M. G. Tomlinson I. (2017). Validation of recently proposed colorectal cancer susceptibility gene variants in an analysis of families and patients—a systematic review. *Gastroenterology* 152 75–77. 27713038 10.1053/j.gastro.2016.09.041 PMC5860724

[B5] Chubb D. Broderick P. Dobbins S. E. Frampton M. Kinnersley B. Penegar S. (2016). Rare disruptive mutations and their contribution to the heritable risk of colorectal cancer. *Nat. Commun.* 7:11883. 10.1038/ncomms11883 27329137 PMC4917884

[B6] Chubb D. Broderick P. Frampton M. Kinnersley B. Sherborne A. Penegar S. (2015). Genetic diagnosis of high-penetrance susceptibility for colorectal cancer (CRC) is achievable for a high proportion of familial crc by exome sequencing. *J. Clin. Oncol.* 33 426–432. 25559809 10.1200/JCO.2014.56.5689

[B7] Ciavarella M. Miccoli S. Prossomariti A. Pippucci T. Bonora E. Buscherini F. (2018). Somatic APC mosaicism and oligogenic inheritance in genetically unsolved colorectal adenomatous polyposis patients. *Eur. J. Hum. Genet.* 26 387–395. 29367705 10.1038/s41431-017-0086-y PMC5839046

[B8] Clarke E. Green R. C. Green J. S. Mahoney K. Parfrey P. S. Younghusband H. B. (2012). Inherited deleterious variants in GALNT12 are associated with CRC susceptibility. *Hum. Mutat.* 33 1056–1058. 10.1002/humu.22088 22461326

[B9] Djursby M. Wadt K. Frederiksen J. H. Madsen M. B. Berchtold L. A. Hasselby J. P. (2020). A rare missense variant in APC interrupts splicing and causes AFAP in two danish families. *Hered. Cancer Clin. Pract.* 3 4–9. 10.1186/s13053-020-00140-3 PMC7140378 32292534

[B10] Dominguez-Valentin M. Nakken S. Tubeuf H. Vodak D. Ekstrøm P. O. Nissen A. M. (2018). Identification of genetic variants for clinical management of familial colorectal tumors. *BMC Med. Genet.* 19:26. 10.1186/s12881-018-0533-9 29458332 PMC5819082

[B11] Drost M. Zonneveld J. B. M. van Hees S. Rasmussen L. J. Hofstra R. M. W. de Wind N. (2012). A rapid and cell-free assay to test the activity of lynch syndrome-associated MSH2 and MSH6 missense variants. *Hum. Mutat.* 33 488–494. 10.1002/humu.22000 22102614

[B12] Espenschied C. R. LaDuca H. Li S. McFarland R. Gau C. L. Hampel H. (2017). Multigene panel testing provides a new perspective on Lynch syndrome. *J. Clin. Oncol.* 35 2568–2575. 10.1200/JCO.2016.71.9260 28514183 PMC7186580

[B13] Evans D. R. Venkitachalam S. Revoredo L. Dohey A. T. Clarke E. Pennell J. J. (2018). Evidence for GALNT12 as a moderate penetrance gene for colorectal cancer. *Hum. Mutat.* 39 1092–1101. 10.1002/humu.23549 29749045 PMC6043371

[B14] Fievet A. Mouret-fourme E. Colas C. De Pauw A. Stoppa-lyonnet D. Buecher B. (2019). Prevalence of pathogenic variants of FAN1 in more than 5000 patients assessed for genetic predisposition to colorectal, breast, ovarian, or other cancers. *Gastroenterology* 156 1919–1920. 30639725 10.1053/j.gastro.2019.01.003

[B15] Frampton M. J. E. Law P. Litchfield K. Morris E. J. Kerr D. Turnbull C. (2016). Implications of polygenic risk for personalised colorectal cancer screening. *Ann. Oncol.* 27 429–434. 10.1093/annonc/mdv540 26578737

[B16] Gammie A. E. Erdeniz N. Beaver J. Devlin B. Nanji A. Rose M. D. (2007). Functional characterization of pathogenic human MSH2 missense mutations in *Saccharomyces cerevisiae*. *Genetics* 177 707–721. 10.1534/genetics.107.071084 17720936 PMC2034637

[B17] Guda K. Moinova H. He J. Jamison O. Ravi L. Natale L. (2009). Inactivating germ-line and somatic mutations in polypeptide N-acetylgalactosaminyltransferase 12 in human colon cancers. *Proc. Natl. Acad. Sci. U.S.A.* 106 12921–12925. 19617566 10.1073/pnas.0901454106 PMC2722285

[B18] Hansen M. F. Johansen J. Sylvander A. E. Bjørnevoll I. Talseth-Palmer B. A. Lavik L. A. S. (2017). Use of multigene-panel identifies pathogenic variants in several CRC-predisposing genes in patients previously tested for Lynch Syndrome. *Clin. Genet.* 92 405–414. 10.1111/cge.12994 28195393

[B19] Heald B. Hampel H. Church J. Dudley B. Hall M. J. Mork M. E. (2020). Collaborative group of the americas on inherited gastrointestinal cancer position statement on multigene panel testing for patients with colorectal cancer and/or polyposis. *Fam. Cancer* 19 223–239. 10.1007/s10689-020-00170-9 32172433 PMC7326311

[B20] Houlleberghs H. Dekker M. Lantermans H. Kleinendorst R. Dubbink H. J. Hofstra R. M. W. (2016). Oligonucleotide-directed mutagenesis screen to identify pathogenic Lynch syndrome-associated MSH2 DNA mismatch repair gene variants. *Proc. Natl. Acad. Sci. U.S.A.* 113 4128–4133. 10.1073/pnas.1520813113 26951660 PMC4839441

[B21] Jagmohan-Changur S. Poikonen T. Vilkki S. Launonen V. Wikman F. Orntoft T. F. (2003). EXO1 variants occur commonly in normal population: evidence against a role in hereditary nonpolyposis colorectal cancer. *Cancer Res.* 63 154–158. 12517792

[B22] Krishnan R. Boddapati N. Mahalingam S. (2018). Interplay between human nucleolar GNL1 and RPS20 is critical to modulate cell proliferation. *Sci. Rep.* 8 1–16. 30061673 10.1038/s41598-018-29802-y PMC6065441

[B23] LaDuca H. Polley E. C. Yussuf A. Hoang L. Gutierrez S. Hart S. N. (2019). A clinical guide to hereditary cancer panel testing: evaluation of gene-specific cancer associations and sensitivity of genetic testing criteria in a cohort of 165,000 high-risk patients. *Genet. Med.* 22 407–415. 10.1038/s41436-019-0633-8 31406321 PMC7000322

[B24] Li H. (2013). Aligning sequence reads, clone sequences and assembly contigs with BWA-MEM. *arXiv* [Preprint]. 10.6084/M9.FIGSHARE.963153.V1

[B25] Lorca V. Rueda D. Martín-Morales L. Poves C. Fernández-Aceñero M. J. Ruiz-Ponte C. (2017). Role of GALNT12 in the genetic predisposition to attenuated adenomatous polyposis syndrome. *PLoS One* 12:e0187312. 10.1371/journal.pone.0187312 29095867 PMC5667827

[B26] Martin-Morales L. Rofes P. Diaz-Rubio E. Llovet P. Lorca V. Bando I. (2018). Novel genetic mutations detected by multigene panel are associated with hereditary colorectal cancer predisposition. *PLoS One* 13:e0203885. 10.1371/journal.pone.0203885 30256826 PMC6157886

[B27] McKenna A. Hanna M. Banks E. Sivachenko A. Cibulskis K. Kernytsky A. (2010). The Genome Analysis Toolkit: a MapReduce framework for analyzing next-generation DNA sequencing data. *Genome Res.* 20 1297–1303. 10.1101/gr.107524.110.20 20644199 PMC2928508

[B28] Morak M. Massdorf T. Sykora H. Kerscher M. Holinski-Feder E. (2011). First evidence for digenic inheritance in hereditary colorectal cancer by mutations in the base excision repair genes. *Eur. J. Cancer* 47 1046–1055. 21195604 10.1016/j.ejca.2010.11.016

[B29] Mork M. E. You Y. N. Ying J. Bannon S. A. Lynch P. M. Rodriguez-Bigas M. A. (2015). High prevalence of hereditary cancer syndromes in adolescents and young adults with colorectal cancer. *J. Clin. Oncol.* 33 3544–3549. 10.1200/JCO.2015.61.4503 26195711 PMC4979241

[B30] Mur P. Jemth A. S. Bevc L. Amaral N. Navarro M. Valdés-Mas R. (2018). Germline variation in the oxidative DNA repair genes NUDT1 and OGG1 is not associated with hereditary colorectal cancer or polyposis. *Hum. Mutat.* 39 1214–1225. 10.1002/humu.23564 29900613

[B31] Nieminen T. T. O’Donohue M. F. Wu Y. Lohi H. Scherer S. W. Paterson A. D. (2014). Germline mutation of RPS20, encoding a ribosomal protein, causes predisposition to hereditary nonpolyposis colorectal carcinoma without DNA mismatch repair deficiency. *Gastroenterology* 147 595–598. 24941021 10.1053/j.gastro.2014.06.009 PMC4155505

[B32] Nilbert M. Wikman F. P. Hansen T. V. O. Krarup H. B. Örntoft T. F. Nielsen F. C. (2009). Major contribution from recurrent alterations and MSH6 mutations in the Danish Lynch syndrome population. *Fam. Cancer* 8 75–83. 10.1007/s10689-008-9199-3 18566915

[B33] Okkels H. Lagerstedt-robinsson K. Wikman F. P. Hansen T. O. Lolas I. Lindberg L. J. (2019). Detection of PMS2 mutations by screening hereditary nonpolyposis colon cancer families from denmark and sweden. *Genet. Test. Mol. Biomarkers* 23 688–695. 31433215 10.1089/gtmb.2018.0316

[B34] Pearlman R. Frankel W. Swanson B. Zhao W. Yilmaz A. Miller K. (2017). Prevalence and spectrum of germline cancer susceptibility gene mutations among patients with early-onset colorectal cancer. *JAMA Oncol.* 3 464–471. 10.1111/mcn.12149 27978560 PMC5564179

[B35] Pedersen B. S. Quinlan A. R. (2018). Mosdepth: quick coverage calculation for genomes and exomes. *Bioinformatics* 34 867–868. 10.1093/bioinformatics/btx699 29096012 PMC6030888

[B36] Plon S. E. Eccles D. M. Easton D. Foulkes W. D. Genuardi M. Greenblatt M. S. (2008). Sequence variant classification and reporting: recommendations for improving the interpretation of cancer susceptibility genetic test results. *Hum. Mutat.* 29 1282–1291. 10.1002/humu.20880 18951446 PMC3075918

[B37] Poplin R. Ruano-Rubio V. DePristo M. A. Fennell T. J. Carneiro M. O. Auwera G. A. V. (2017). Scaling accurate genetic variant discovery to tens of thousands of samples. *bioRxiv* [Preprint]. 10.1101/201178

[B38] Rath A. Mishra A. Ferreira V. D. Hu C. Omerza G. Kelly K. (2019). Functional interrogation of Lynch syndrome-associated MSH2 missense variants via CRISPR-Cas9 gene editing in human embryonic stem cells. *Hum. Mutat.* 40 2044–2056. 31237724 10.1002/humu.23848 PMC6810757

[B39] Richards S. Aziz N. Bale S. Bick D. Das S. Gastier-foster J. (2015). Standards and guidelines for the interpretation of sequence variants: a joint consensus recommendation of the american college of medical genetics and genomics and the association for molecular pathology. *Genet. Med.* 17 405–424. 10.1038/gim.2015.30.Standards 25741868 PMC4544753

[B40] Robinson J. T. Thorvaldsdóttir H. Winckler W. Guttman M. Lander E. S. Getz G. (2011). Integrative genome viewer. *Nat. Biotechnol.* 29 24–26. 10.1038/nbt.1754.Integrative 21221095 PMC3346182

[B41] Rohlin A. Rambech E. Kvist A. Törngren T. Eiengård F. Lundstam U. (2017). Expanding the genotype–phenotype spectrum in hereditary colorectal cancer by gene panel testing. *Fam. Cancer* 16 195–203. 10.1007/s10689-016-9934-0 27696107 PMC5357488

[B42] Rohlin A. Zagoras T. Nilsson S. Lundstam U. Wahlström J. Hultén L. (2014). A mutation in POLE predisposing to a multi-tumour phenotype. *Int. J. Oncol.* 45 77–81. 10.3892/ijo.2014.2410 24788313 PMC4079162

[B43] Schubert S. A. Morreau H. de Miranda N. F. C. C. van Wezel T. (2019). The missing heritability of familial colorectal cancer. *Mutagenesis* 35 221–231. 10.1093/mutage/gez027 31605533 PMC7352099

[B44] Seguí N. Mina L. B. Lázaro C. Sanz-Pamplona R. Pons T. Navarro M. (2015). Germline mutations in FAN1 cause hereditary colorectal cancer by impairing DNA repair. *Gastroenterology* 149 563–566. 26052075 10.1053/j.gastro.2015.05.056

[B45] Seguí N. Pineda M. Navarro M. Lázaro C. Brunet J. Infante M. (2014). GALNT12 is not a major contributor of familial colorectal cancer type X. *Hum. Mutat.* 35 50–52. 10.1002/humu.22454 24115450

[B46] Smith C. G. West H. Harris R. Idziaszczyk S. Maughan T. S. Kaplan R. (2013). Role of the oxidative DNA damage repair gene OGG1 in colorectal tumorigenesis. *J. Natl. Cancer Inst.* 105 1249–1253. 10.1093/jnci/djt183 23852950

[B47] Stoffel E. Koeppe E. Everett J. Ulintz P. Kiel M. Osborne J. (2018). Germline Genetic Features of Young Individuals with Colorectal Cancer. *Gastroenterology* 154 897–905. 10.1016/j.physbeh.2017.03.040 29146522 PMC5847426

[B48] Talseth-Palmer B. A. Bauer D. C. Sjursen W. Evans T. J. Mcphillips M. Proietto A. (2016). Targeted next-generation sequencing of 22 mismatch repair genes identifies Lynch syndrome families. *Cancer Med.* 5 929–941. 10.1002/cam4.628 26811195 PMC4864822

[B49] Terradas M. Capellá G. Valle L. (2020). Dominantly inherited hereditary nonpolyposis colorectal cancer not caused by MMR genes. *J. Clin. Med.* 9:1954. 10.3390/jcm9061954 32585810 PMC7355797

[B50] Thompson B. A. Snow A. K. Koptiuch C. Kohlmann W. K. Mooney R. Johnson S. (2020). A novel ribosomal protein S20 variant in a family with unexplained colorectal cancer and polyposis. *Clin. Genet.* 97 943–944. 10.1111/cge.13757 32424863

[B51] Vande Perre P. Siegfried A. Corsini C. Bonnet D. Toulas C. Hamzaoui N. (2019). Germline mutation p.*N363K* in POLE is associated with an increased risk of colorectal cancer and giant cell glioblastoma. *Fam. Cancer* 18 173–178. 30368636 10.1007/s10689-018-0102-6

[B52] Vaughn C. P. Robles J. Swensen J. J. Miller C. E. Lyon E. Mao R. (2010). Clinical analysis of PMS2: mutation detection and avoidance of pseudogenes. *Hum. Mutat.* 31 588–593. 10.1002/humu.21230 20205264

[B53] Venkatachalam R. Ligtenberg M. J. L. Hoogerbrugge N. Schackert H. K. Görgens H. Hahn M. M. (2010). Germline epigenetic silencing of the tumor suppressor gene PTPRJ in early-onset familial colorectal cancer. *Gastroenterology* 139 2221–2224. 10.1053/j.gastro.2010.08.063 21036128

[B54] Wang D. Zhang Z. Li Y. Xu C. Yu Y. Li M. (2019). Adenomatous polyposis coli gene mutations in 22 Chinese pedigrees with familial adenomatous polyposis. *Med. Sci. Monit.* 25 3796–3803. 10.12659/MSM.913911 31113927 PMC6542301

[B55] Weitzel J. N. Chao E. C. Nehoray B. Van L. R. Pesaran T. Rybak C. (2018). Somatic TP53 variants frequently confound germline testing results. *Genet Med.* 20 809–816. 10.1038/gim.2017.196.Somatic 29189820 PMC5976505

[B56] Weren R. D. A. Ligtenberg M. J. L. Kets C. M. De Voer R. M. Verwiel E. T. P. Spruijt L. (2015). A germline homozygous mutation in the base-excision repair gene NTHL1 causes adenomatous polyposis and colorectal cancer. *Nat. Genet.* 47 668–671. 25938944 10.1038/ng.3287

[B57] Yurgelun M. B. Kulke M. H. Fuchs C. S. Allen B. A. Uno H. Hornick J. L. (2017). Cancer susceptibility gene mutations in individuals with colorectal cancer. *J. Clin. Oncol.* 35 1086–1095. 10.1200/JCO.2016.71.0012 28135145 PMC5455355

